# Nanoworld of terahertz magnetism opening new frontiers

**DOI:** 10.1016/j.newton.2026.100521

**Published:** 2026-09-08

**Authors:** Alexey V. Kimel, Boris A. Ivanov, Dmytro Afanasiev

**Affiliations:** 1 Radboud University, Heyendaalseweg 135, 6525 AJ Nijmegen, the Netherlands; 2 V.G. Baryakhtar Institute of Magnetism of the NASU, Vernadsky Av. 36B, 03142 Kyiv, Ukraine

**Keywords:** antiferromagnets, altermagnets, terahertz spectroscopy, spin dynamics, ultrafast magnetism, magneto-optics, opto-magnetism, magnonics

## Abstract

The quest to understand magnetic phenomena at ever-smaller length scales and faster time scales has long driven magnetism research, yielding numerous breakthroughs from the Nobel Prize-winning discovery of giant magnetoresistance to advanced applications. Understanding (sub)picosecond (THz) spin dynamics at the nanoscale has further motivated the development of unique instrumentation for nanofabrication and scanning probe microscopy, as well as multi-billion-dollar multinational projects such as X-ray free-electron lasers. In this perspective, we describe fundamental principles for exciting and detecting coherent spin-wave excitations—magnons—at THz frequencies and nanometer wavelengths without relying on X-rays or nanopatterning techniques. Our idea is to show that there is a practical approach to open up new avenues for exploring ultrafast magnetism at the nanoscale using a conventional femtosecond laser source, accessible in virtually any laboratory.

## Accessible overview

The quest to understand magnetic phenomena at ever-smaller length scales and faster time scales has long driven magnetism research, leading to numerous breakthroughs in both fundamental research and technology. These include the Nobel Prize-winning discovery of giant magnetoresistance, advances in topological magnetism, and the exploration of ever-faster spin switching. Understanding femtosecond and picosecond (THz) spin dynamics at the nanoscale has been a key motivation behind the development of specialized instrumentation for nanofabrication and scanning probe microscopy, as well as multi-billion-dollar multinational projects such as X-ray free-electron lasers. In this perspective, we describe the fundamental principles for exciting and detecting coherent spin-wave excitations—magnons—at THz frequencies and nanometer wavelengths without relying on X-rays or nanopatterning techniques. Our aim is to show that there a specific approach and class of materials can open new avenues for exploring ultrafast coherent magnetism at the nanoscale in virtually any laboratory equipped with a conventional femtosecond laser source. This perspective offers the very first comprehensive review summarizing optical, and thus widely accessible, approaches to excite and detect sub-100-nm spin waves in antiferromagnets with sup-ps temporal resolution. We explain the main physical principles of interaction governing the interaction of light with THz antiferromagnetic spin waves at nanometer wavelengths, highlight novel and intriguingly exotic regimes anticipated in propagation of such THz waves, and discuss how these waves can facilitate further progress in fundamental research on THz nanomagnetism.

## Graphical abstract

**Figure:**
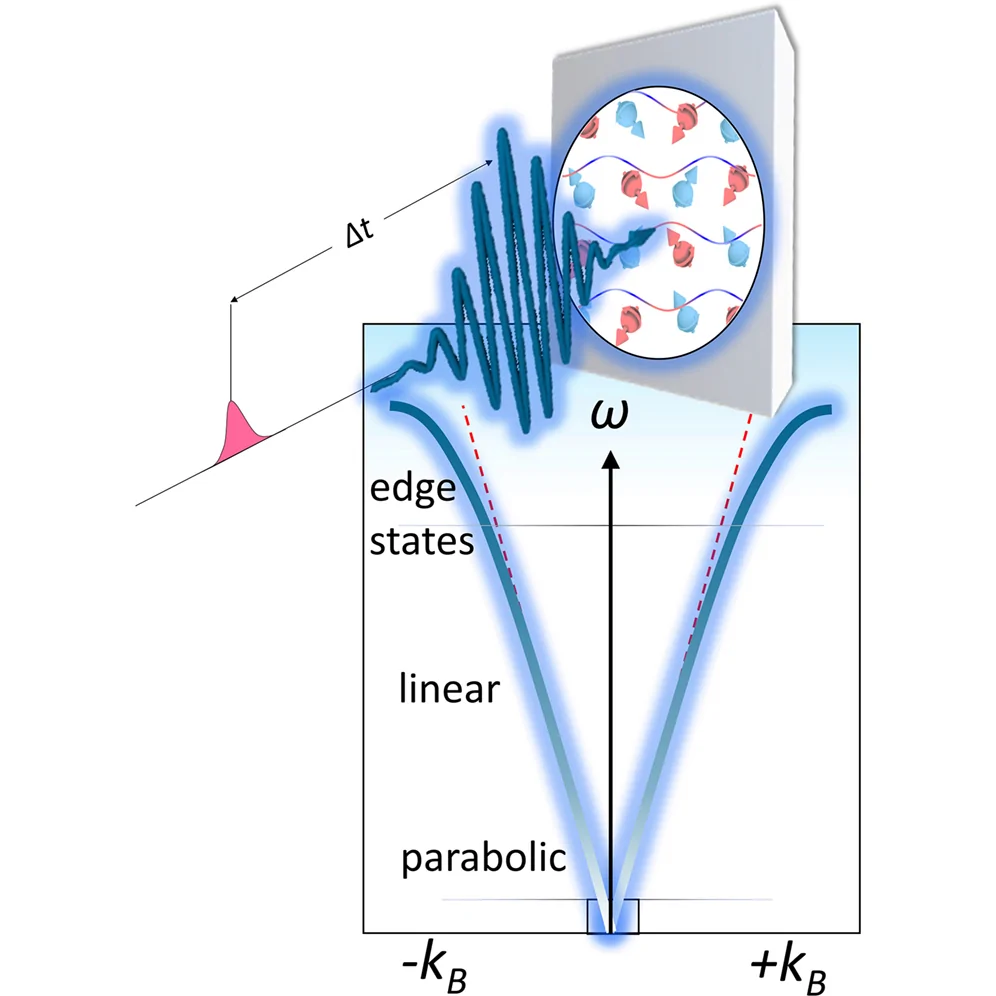

## Teaser

Understanding ultrafast magnetism at the nanoscale has been a long-standing challenge. In this first comprehensive review, Kimel et al. summarize optical, and thus widely accessible, approaches to excite and detect sub-100-nm spin waves in antiferromagnets with sup-ps temporal resolution. They explain light interaction with THz antiferromagnetic spin waves at nanometer wavelengths, highlight exotic propagation regimes, and discuss how these waves can facilitate further progress in fundamental research on THz nanomagnetism.

## Introduction

For more than a decade, it has been anticipated that once magnonics enables the excitation, control, and detection of coherent magnons (quanta of spin waves) with nanometer wavelengths and terahertz (THz) frequencies, it will open the door to a technological breakthrough.^1^ In particular, magnonics was believed to be a solution for low-dissipation computing with integrated memory, scalable to the nanometer regime, and capable of processing data at terabit-per-second rates comparable to modern telecommunication.^2^ However, studying excitation, control, and detection of such coherent spin waves is, above all, interesting from a fundamental point of view.

Magnetism is arguably the most prominent manifestation of quantum mechanics on Earth. The quantum-mechanical exchange interaction, which is responsible for the very existence of magnetic order, is so strong that it can preserve magnetic order up to remarkably high temperatures, which for iron and cobalt are very close to their melting points above 1,000 K. Despite this quantum origin, the development of theory in magnetism was greatly spurred by introduction of the approximation of continuous medium, which, instead of analyzing quantum-mechanical dynamics of individual spins, treats a magnet as a continuous medium described by slowly varying in space macroscopic, i.e., classical, parameters—a net magnetization **M** for ferromagnets and antiferromagnetic Néel vector **L** for an antiferromagnet.^3^ The footprints of the quantum-mechanical nature of magnetism still remain within this approach, and in particular, the high symmetry of exchange interaction shows up in nearly isotropic length of the net magnetization **M** and/or the Néel vector **L**. The validity of the continuous medium approximation, however, requires that the characteristic length scale remains much larger than the interatomic spacing. Hence, as the spin-wave wavelength decreases, the approximation of continuous media must inevitably break down.^4^ Studying magnons with nanometer wavelength and THz frequencies promises to reveal truly quantum magnetism.

Magnons in magnetic materials are described by a dispersion relation *ω*(*k*), which links their frequency *ω* to the wavevector k=2πλ, with *λ* the magnon wavelength. In the conventional approach, magnons are excited using microwave antennas that emit spin waves at a chosen frequency and wavevector. However, accessing magnons with nanometre-scale wavelengths would require fabricating antennas of comparable dimensions. Moreover, generating and handling THz-frequency signals on a chip remains a major technological challenge.^5^ Similar problems would also be valid for detection.

While the vast majority of fundamental research in magnonics has so far been focused on ferromagnets and ferrimagnets,^6^ we demonstrate here that antiferromagnets—the largest yet least explored class of magnets—offer elegant solutions for exciting and detecting THz magnons with nanometer wavelengths, thereby opening a new avenue in magnetism research. The key ingredient of these solutions is femtosecond (fs) laser pulse.

## Antiferromagnetic magnons and ultrashort pulses of light

**Figure 1:**
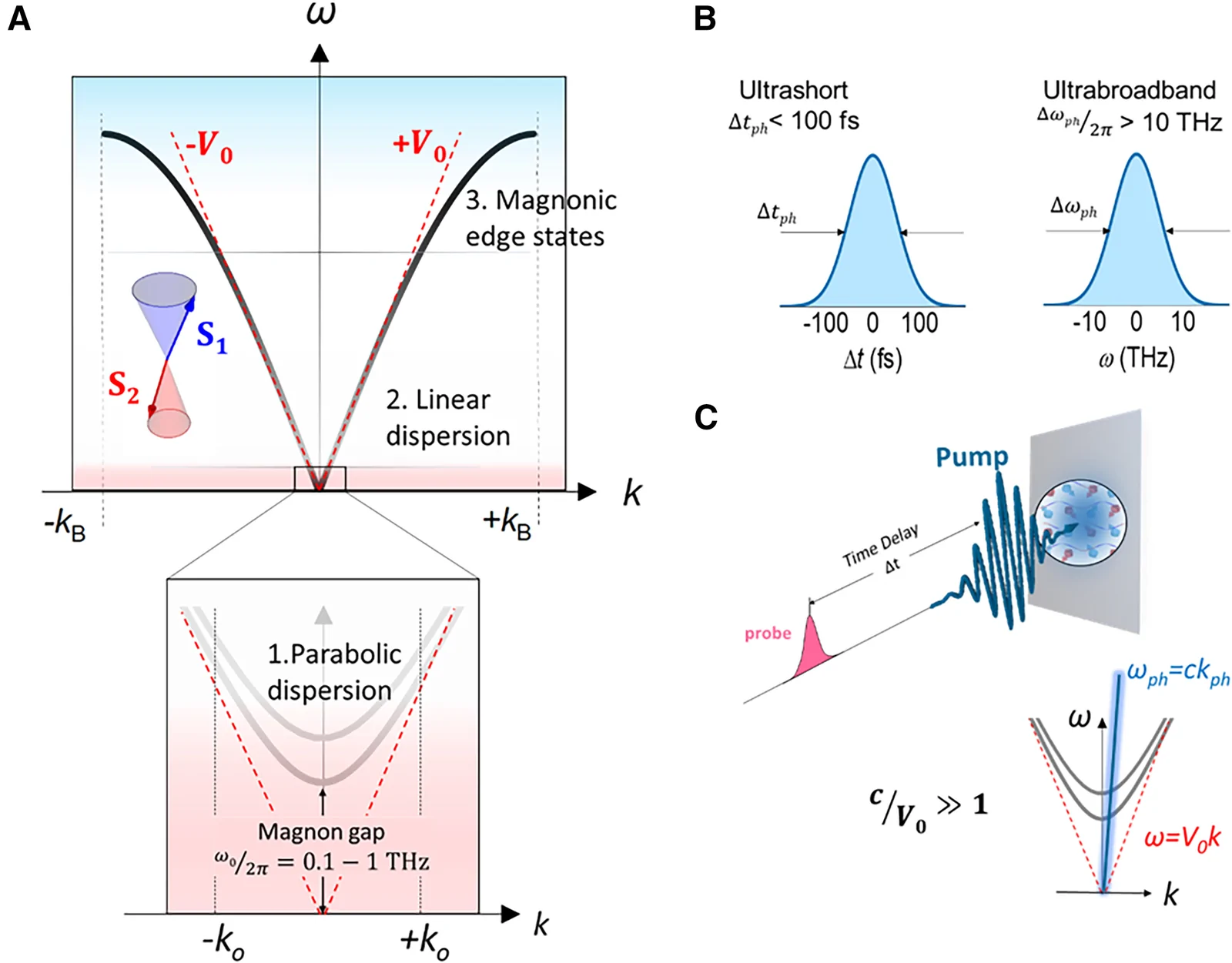
Antiferromagnetic magnons and ultrashort pulses of light (A) Schematics of the magnon dispersion of an antiferromagnet from the center (*k* = 0) to the edge of the Brillouin zone (*k* = *k*_B_). Three distinct regimes of magnon propagation can be identified: (1) the low-wavevector regime characterized by parabolic dispersion, (2) the intermediate regime with linear dispersion corresponding to exchange-dominated dynamics, and (3) the regime of magnonic edge states emerging near the Brillouin-zone boundary. *V*_0_ and *ω*_0_ are the limiting velocity of the magnons and the magnon gap, respectively. k0=ω0V0 defines a minimal magnon momentum above which the dispersion relation *ω*(*k*) acquires a characteristic linear form (B) Temporal profile of the optical pulse with duration Δ*t*_*ph*_ together with its spectral bandwidth profile Δ*ω*_*ph*_. (C) A schematic of a time-resolved pump-probe experiment used to excite coherent magnons in antiferromagnets. The inset shows a problem of linear momentum mismatch that leads to dominant excitation of zone-center (*k* = 0) magnons characterized by the smallest frequency and group velocities.

The number of magnon branches in an antiferromagnet is set by the number of magnetic ions in the unit cell. While complex materials may host multiple branches, a minimal two-sublattice antiferromagnet comprising spins **S**_1_ and **S**_2_ coupled by exchange interactions that favor antiparallel alignment exhibits two magnon modes, as shown in Figure 1A. Their dispersion away from the Brillouin zone edge can be expressed as

(Equation 1) $$\begin{array}{cc} \;\omega \left(k\right)=\sqrt{\omega_{0,i}^{2}+{\left(V_{0}k\right)}^{2}} & \end{array}\text{,}$$

which clearly resembles the energy-momentum relation for relativistic particles, where the first term, known as the magnon gap *ω*_0,*i*_ is specific for each of the magnon branches (*i* = 1,2). It plays the role of rest energy of the particle and the characteristic velocity *V*_0_ plays the role of the speed of light. For sufficiently large wavevectors k>k0,i=ω0,iV0, the magnon frequency *ω* becomes a linear function of *k* that corresponds to the case of ultra-relativistic particles. The exchange-limited value $V_{0}=\frac{2J_{exc}Sa}{\hbar }$ typically exceeds 10 km/s, where *J*_*exc*_ is the intersublattice exchange constant, *S* is the spin magnitude, and *a* is the lattice constant. In the case of *k* > *k*_0,*i*_, the spin-wave group velocity $V_{k}=\frac{\partial \omega \left(k\right)}{\partial k}$ is nearly independent of the magnon momentum *k*, rapidly reaching the largest possible value *V*_0_. This behavior of magnons in antiferromagnets is fundamentally different from that of ferromagnets, where the magnon dispersion is intrinsically quadratic and the group velocity *V*_*k*_∝*k* increases gradually with *k*, reaching its limiting value only near the Brillouin zone edge kB=πa≫k0. Moreover, while in ferromagnets, the magnon gap frequencies *ω*_0_ are typically set by magnetic anisotropies and lie in the gigahertz (GHz) range, in antiferromagnets the strong intersublattice exchange interaction pushes the magnon energies into the THz regime. As a result, magnons of the same wavelength (or wavenumber *k*) in antiferromagnets not only have far higher frequencies than in ferromagnets, but also propagate much faster.

Fs light pulses (Δ*t*_*ph*_< 100 fs) are among the shortest stimuli in condensed matter physics and possess spectral bandwidths Δ*ω*_*ph*_/2*π* > 10 THz, sufficient to excite magnons across the full Brillouin zone in antiferromagnets, see Figure 1B. This spectral overlap made fs pulses an indispensable tool for studying coherent spin dynamics in antiferromagnets.^7^ By selectively perturbing microscopic interactions such as the exchange or anisotropy, the optical excitation generates ultrashort effective optomagnetic fields approaching 1 T, capable of driving large-amplitude spin precession and transiently reorienting the Néel vector. In time-resolved pump-probe experiments, such pulses enable coherent control of the antiferromagnetic Néel vector **L** and direct tracking of its dynamics down to the fs timescale (see Figure 1C). However, due to the large mismatch between photon and magnon momenta, conventional approaches allow efficient optical excitation and detection only of low-wavenumber (*k* ≃ 0) magnons, corresponding to quasi-uniform spin excitations characterized not only by the lowest frequencies but also by negligible group velocities. A comparable momentum-mismatch problem has been successfully addressed in seminal studies of ultrafast lattice dynamics.^8^^,^^9^^,^^10^^,^^11^ The solutions pioneered there, naturally, have also inspired the development of similar approaches in the application of ultrafast dynamics of spins at high *k*.

## Excitation of coherent THz spin waves by absorption gradients and Bragg-inspired magneto-optical detection

A straightforward approach to overcoming the photon-magnon momentum mismatch is to spatially confine the optical excitation. By localizing the action of the optical field to a small region, spin waves with momenta up to Δ*k*∼1/Δ*x* can be generated, where Δ*x* is the size of the laser-excited region. Focusing the light offers the most straightforward realization of this approach, producing spin waves with higher momenta (i.e., shorter wavelengths). A simple estimate shows that, to reach the linear part of the dispersion and thereby access the ultra-relativistic regime of spin-wave propagation, the confinement must satisfy Δ*x*≪2*πV*_0_/*ω*_0_. Considering the spin-wave gap frequencies, *ω*_0_/2*π* = 0.1–10 THz, and velocities of the order of 10 km/s, the required confinement is 10–100 nm, which is well below the diffraction limit for visible light.

**Figure 2:**
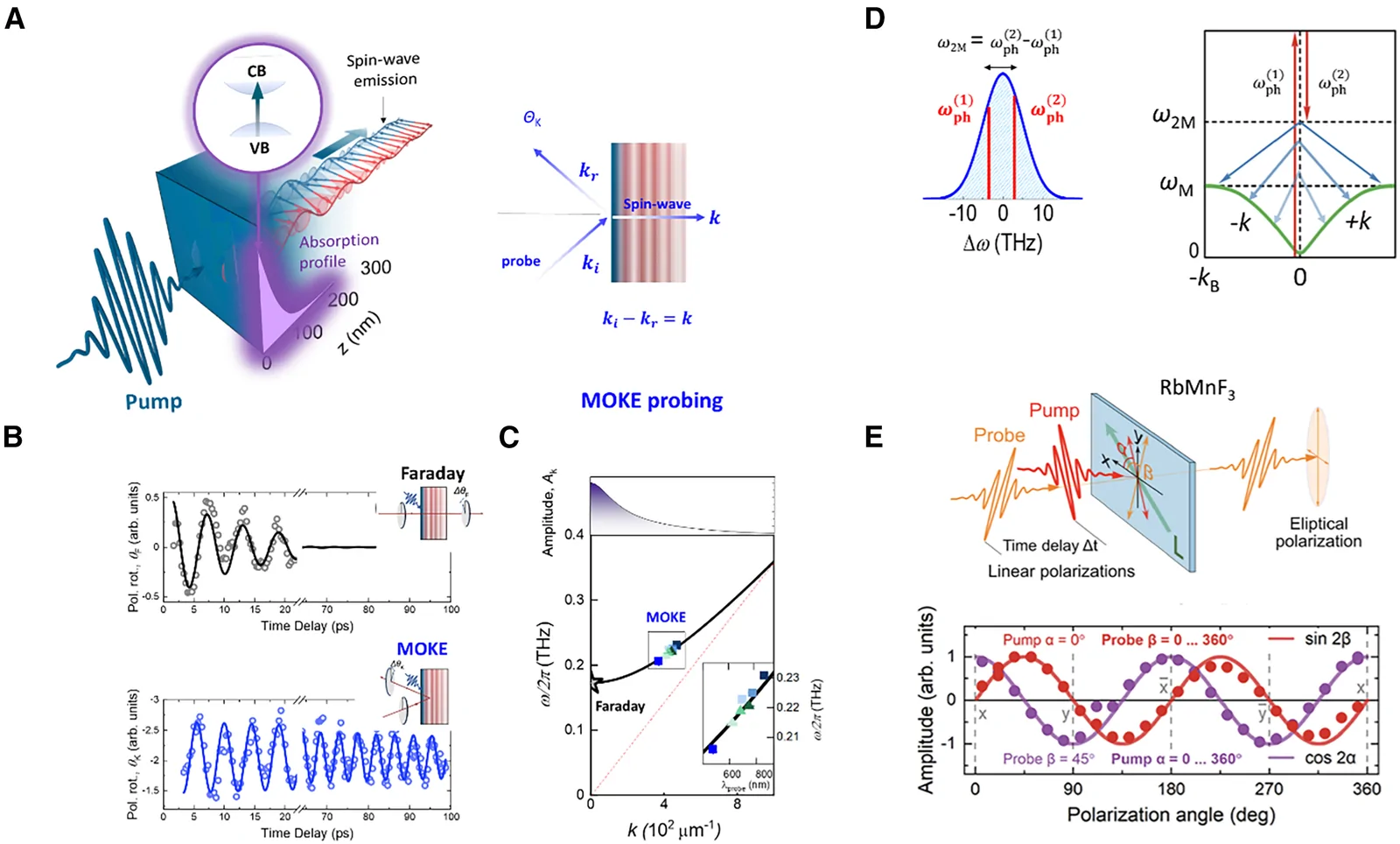
Excitation of high-*k* nanoscale antiferromagnetic coherent magnons by light (A) (Left) Schematic illustration of absorption-gradient-driven optical pumping leading to the emission of a broadband wavepacket of coherent magnons following above-bandgap excitation. (Right) Bragg-inspired, wavevector-selective *k* probing of coherent magnons via measurement of the polarization rotation *θ*_k_ induced by the magneto-optical Kerr effect (MOKE). (B) Comparison of pump-induced coherent spin dynamics in antiferromagnetic DyFeO_3_ measured via (top) Faraday rotation, sensitive to spin waves with *k* = 0, and (bottom) MOKE, sensitive to spin waves with finite wavevector *k* defined by the phase-matching condition *k* = *k*_i_ − *k*_r_, where *k*_i_ and *k*_r_ are the wavevectors of the incident and reflected probe light, respectively. (C) Mapping of the magnon dispersion in DyFeO_3_ via probe-wavelength-selective detection of spectral magnon components *A*_k_ excited by above-band-gap pumping. The top image demonstrates the spectral distribution of the components. Adapted from Hortensius et al.^12^ (D) Pairwise two-magnon (2M) excitation of coherent magnons across the entire Brillouin zone by two photons with frequencies differing by 2*ω*_*M*_ = $\omega_{ph}^{\left(2\right)}$ − $\omega_{ph}^{\left(1\right)}$ simultaneously present within the broadband femtosecond pulse. (E) (Top) Experimental geometry used for excitation and detection of coherent 2M states. A linearly polarized optical pump pulse excites spin dynamics, which is detected via the transient ellipticity of the optical probe pulse time delayed by Δ*t*. The projection of the antiferromagnetic vector L onto the *xy* sample plane is shown. (Bottom) Optical selection rules for excitation and detection of the 2M mode in RbMnF_3_. The pump and probe polarization angles are measured relative to the crystallographic *x* axis. Adapted from Formisano et al.^16^

This limitation can be overcome by exploiting the distinctive features of above-band-gap optical absorption in antiferromagnets. Many of them are wide-band-gap insulators, typically with gaps corresponding to photon energies in the ultraviolet spectral range. Under above-band-gap excitation, the penetration depth of light can be employed to localize the optical perturbation on subwavelength scales, thereby surpassing the limitations of the diffraction limit (see left image in Figure 2A) Indeed, the above-band-gap absorption has recently been exploited to generate broadband spin-wave excitations in antiferromagnetic orthoferrites and hematite, confining the effects of optical fields on spins to below 50 nm and producing a highly inhomogeneous profile of spin excitation.^12^^,^^13^^,^^14^^,^^15^ This inhomogeneity enables the emission of broadband coherent THz spin-wave packets with bandwidths exceeding 0.1 THz and group velocities above 12 km/s, significantly surpassing the velocities of sound waves and thus the velocity of heat propagation as well.

The magneto-optical Kerr effect (MOKE) offers a sensitive way to probe the spatially inhomogeneous dynamics of propagating spin waves (see right image in Figure 2A). When above-band-gap absorption creates a strongly nonuniform spin excitation along the surface normal *z*, it launches spin waves that propagate into the sample along the same direction. Even in a fully compensated antiferromagnet, the spin-wave oscillations are associated with dynamic magnetization proportional to $L\times \frac{dL}{dt}$.^17^ In the case of a monochromatic plane spin wave at the frequency *ω*, the magnetization reads:

(Equation 2) $$M\left(z,t\right)=M_{m}\left(\omega \right)e^{i\left(\omega t-kz\right)}$$

where *M*_*m*_(*ω*) is the spectral amplitude of the magnon mode at the frequency *ω*. If linearly polarized optical wave with the wavevector *k*_*ph*_ is reflected from a sample surface with the magnetization, the MOKE will result in a rotation of the linear polarization of the optical wave by an angle *θ*_*K*_, which is proportional to the time-varying surface magnetization *M*(*z*,*t*). If the magnetization is inhomogeneous and the reflected beam is formed by a surface layer with a thickness *d*, the MOKE signal in the reflected beam for light at normal incidence is a result of integration:

(Equation 3) $$\theta_{K}\propto \int_{0}^{d}dz\;{\;e}^{i2k_{ph}z}\;M_{m}\left(\omega \right)e^{i\left(\omega t-kz\right)}$$

where factor ${\;e}^{i{2k}_{ph}z}$ arises from the phase difference accumulated between the incident and reflected light waves. In general, *k*_*ph*_ is complex, where the imaginary part accounts for the absorption of light in the medium. The integral has a sharp maximum when 2*k*_*ph*_ = *k*, corresponding to the Bragg reflection, which is standard for periodic media. The detection mechanism follows the principles of the Bragg reflection: by tuning the wavelength of the probe light, one adjusts the sensitivity to magnetization modulations of a specific periodicity/wavelength and the corresponding frequency. Figure 2B compares pump-induced spin dynamics in antiferromagnetic DyFeO_3_ measured using the Faraday rotation, which is sensitive to uniform spin waves with *k* = 0, and the MOKE, which is sensitive to spin waves with finite wavevector *k*, clearly showing a difference in the oscillation period of the detected dynamics.

Using *k*-selective Bragg detection—for example, by continuously tuning the probe wavelength or employing broadband probe^18^—and taking advantage of the broadband magnon population generated by above-band-gap excitation, one can measure the spectral amplitudes *M*_*m*_(*ω*) associated with specific wavevectors *k*. By determining their frequencies in time-resolved optical pump-probe measurements, the magnon dispersion can be directly mapped down to wavelengths of less than 100 nm (see Figure 2C).

We note that although above-band-gap optical pumping and *k*-vector selective MOKE detection provide access to nanometer-scale coherent antiferromagnetic magnons, their wavelengths remain significantly larger than the interatomic spacing, ensuring that a macroscopic description remains valid in this regime.

## Two-magnon excitations and detection beyond the Néel vector

If magnons are generated in pairs, light can excite coherent magnons practically over the whole Brillouin zone.^19^^,^^20^^,^^21^^,^^22^ In this process, the incident optical wave with the wavevector **k**_*ph*_ and the frequency *ω*_*ph*_ upon absorption creates two magnons with the wavevectors **k**^(1)^ and **k**^(2)^. Even if *k*_*ph*_≪*k*^(1,2)^, the law of momentum conservation **k**^(1)^+**k**^(2)^ = **k**_*ph*_ can still be satisfied by setting the magnon wavevectors nearly equal in length but opposite in direction. The conservation of energy can simultaneously be satisfied by setting the magnon frequencies (*ω*^(1)^ and *ω*^(2)^) to match that of the absorbed light *ω*_*ph*_, i.e., *ω*^(1)^+*ω*^(2)^ = *ω*_*ph*_. As the density of magnonic states is the largest at the edges of the Brillouin zone, out of all magnons hosted in an antiferromagnet, those with the highest frequency and the shortest wavelength will be excited in the most efficient way. A very similar mechanism for two-magnon (2M) excitation can be achieved, not through absorption, but via light scattering. The process requires two light waves with the frequencies $\omega_{ph}^{\left(1\right)}$ and $\omega_{ph}^{\left(2\right)}$, where $\omega_{ph}^{\left(2\right)}-\omega_{ph}^{\left(1\right)}=\omega^{\left(1\right)}+\omega^{\left(2\right)}$. If one uses sufficiently short, i.e., fs, laser pulses, the width of the laser spectrum will be broader than 2*ω*^(1)^, and hence the laser will be able to impulsively excite pairs of mutually coherent magnons at the two opposite edges of the Brillouin zone (see Figure 2D). This process is called impulsive stimulated Raman scattering by the 2M mode.^23^^,^^24^ From a microscopic perspective, when the pump electric field is aligned along one of the principal crystallographic axes (e.g., the *x* axis), it can resonantly or off-resonantly excite electronic transitions, effectively modifying the electron wavefunctions. These changes directly affect the exchange interaction *J*_*exc*_ between neighboring spins, which determines their correlations. As a result, the time-varying spin correlation function *C*(*t*) along the corresponding crystallographic axis,

(Equation 4) $$C\left(t\right)=2\sum_{i}S_{i}^{\left(x\right)}\left(t\right)S_{i+1}^{\left(x\right)}\left(t\right)\text{,}$$

is impulsively altered and subsequently oscillates at the 2M frequency.

The dynamics of the spin-correlation function corresponding to the dynamics of a pair of mutually coherent magnons at the opposite edges of the Brillouin zone can also be detected optically.^16^ In an otherwise isotropic crystal, where, for the dielectric permittivity in the ground state, one finds *ε*_*xx*_ = *ε*_*yy*_ = *ε*_*zz*_, inequality $\sum_{i}S_{i}^{\left(x\right)}S_{i+1}^{\left(x\right)}\neq \sum_{i}S_{i}^{\left(y\right)}S_{i+1}^{\left(y\right)}$ may, by symmetry, lead to inequality *ε*_*xx*_≠*ε*_*yy*_ and thus the oscillations may be observed through time-resolved measurements of optical birefringence in a pump-probe experiment.

The excitation and detection of nanoscale magnon dynamics corresponding to the (2M) mode have been recently demonstrated in the archetypical cubic Heisenberg antiferromagnet RbMnF_3_,^16^^,^^25^ where measurements of the probe ellipticity arising from optical birefringence provide access to the dynamics of magnons near the edge of the Brillouin zone (see Figure 2E). The strongest oscillations of the probe ellipticity are observed when the pump polarization is aligned along the crystallographic x or y axes (α = 0°or 90°) and the probe polarization is set at β = 45° or 135°. This behavior indicates a pronounced coupling to the crystal symmetry rather than to the Néel vector orientation, which, in this geometry, lies between the *x* and *y* axes. These striking observations highlight the importance of describing the nanoscale dynamics of the shortest-wavelength antiferromagnetic magnons near the Brillouin-zone edge in terms of spin-spin correlation functions, rather than relying solely on a macroscopic description based on the Néel vector.

## Outlook

The ability to generate coherent magnons across a large portion of the Brillouin zone opens new possibilities for both fundamental studies and ultrafast control of magnetism. It enables direct tests of recent predictions of nonreciprocal spin-wave propagation in specific classes of antiferromagnets, offers a powerful dynamic probe of ultrafast magnetic phase transitions, and paves the way to explore the full nonlinear potential of antiferromagnetic media.

### Nonlinear magnonics

The nonlinear interaction of magnons with other magnons and with magnetic nonuniformities—collectively known as magnon scattering—leads to a redistribution of the magnon population across the magnon spectrum.^26^ Antiferromagnets, which host at least two sublattices with oppositely aligned spins (“up” and “down”) and therefore support at least two magnon modes, provide a fertile platform for studying interactions between distinct magnon branches and for exploring the emerging regime of THz nonlinear magnonics. Recent studies of THz light-driven nonlinear dynamics in antiferromagnets have revealed strong interactions between otherwise orthogonal magnon modes, resulting in both the up- and down-conversion of frequencies,^27^^,^^28^ as well as the generation of magnons at entirely new frequencies—analogous to optical sum- and difference-frequency generation of photons.^29^ The observation of up to seventh-order magnon mixing and sixth-harmonic magnon generation using THz two-dimensional coherent spectroscopy demonstrates unprecedented control over complex many-body magnonic states.^30^ Moreover, nonlinear interactions arise not only between different magnon modes but also between magnons and phonons^31^ or excitons,^32^ positioning antiferromagnets as a uniquely powerful platform for nonlinear magnonics. However, the intrinsic momentum mismatch between light and spin excitations has thus far largely confined these interactions to the Brillouin-zone center.

**Figure 3:**
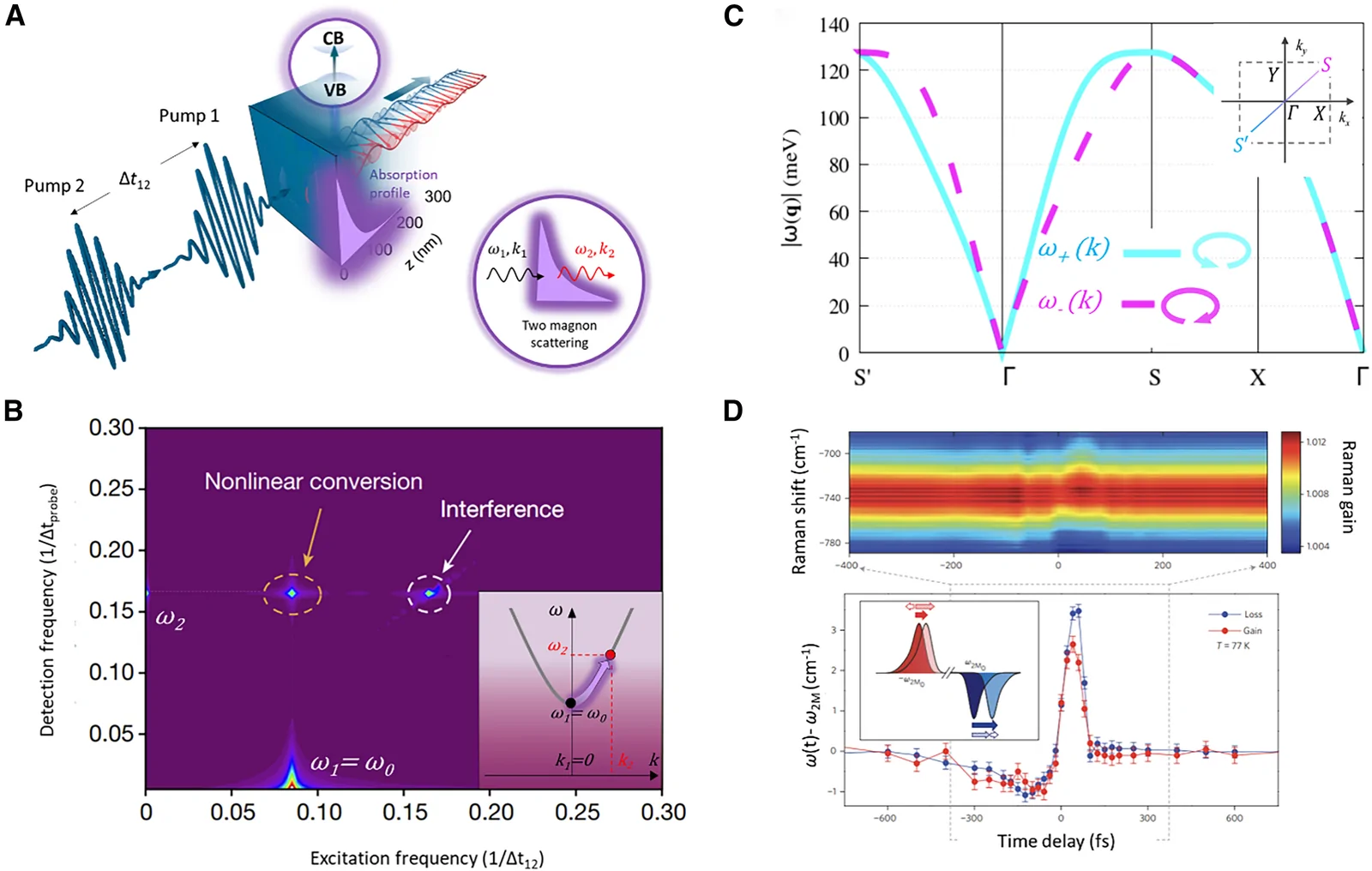
Emerging directions in light-driven THz nanomagnetism in antiferromagnets (A) Schematics of double-pump above-band-gap experiment used to stimulate and probe nonlinear magnon-magnon interactions in antiferromagnetic HoFeO_3_. An initial femtosecond pump pulse (pump 1), with photon energy tuned above the electronic band gap, excites a nonequilibrium spin distribution near the sample surface, within the region defined by the pump penetration depth. This generates coherent magnons with a broad distribution of frequencies across the antiferromagnetic magnon spectrum. A second, time-delayed pump pulse (pump 2) perturbs this nonequilibrium state. This light-induced conversion can be regarded as an optically driven analog of two-magnon (2M) scattering, in which a zone-center magnon (*ω*_1_ = *ω*_0_, *k*_*1*_ = 0) is transformed into propagating magnons (*ω*_2_, *k*_2_) within the nonequilibrium spin population created by the first pump pulse. (B) Two-dimensional coherent magnon spectroscopy frequency map in orthoferrite HoFeO_3_ showing the interconversion of a zone-center (*k* = 0) magnon into finite-momentum (*k* = *k*_2_) magnons. The diagonal peak corresponds to a coherent superposition of magnons (regular interference), while the off-diagonal peak indicates nonlinear mode coupling. The inset schematically illustrates the nonlinear interconversion on the spin-wave spectrum. Adapted from Leenders et al.^15^ (C) Calculated nonreciprocal dispersion of chiral magnons in the altermagnetic candidate RuO_2_ along high-symmetry directions across the full Brillouin zone The inset indicates the positions of the high-symmetry points in reciprocal space. Adapted from Šmejkal et al.^33^ (D) Photoinduced evolution of the 2M Raman mode as a function of pump-probe delay. The 2M frequency, set by the exchange interaction, shifts transiently after ultrafast optical excitation in antiferromagnetic KNiF_3_, directly reflecting light-induced changes of the exchange coupling. Adapted from Batignani et al.^34^

The development of novel optical schemes capable of exciting and probing the dynamics of finite-*k* magnons now promises to overcome the intrinsic momentum mismatch limitation and to establish antiferromagnets as a true nonlinear medium for magnonics of nanoscale THz magnons. Recent experiments have shown that above-band-gap excitation in antiferromagnetic orthoferrite HoFeO_3_ using tailored sequences of fs pulses enables access to a broad region of the spin-wave spectrum and opens pathways for interconversion between finite-momentum (*k* ≠ 0) and zone-center (*k* = 0) magnons.^15^ This light-induced conversion can be regarded as an optically driven analog of 2M scattering, in which a zone-center magnon is transformed into propagating magnons through nonlinear magnon-magnon interactions activated by an ultrafast optical field (see Figure 3A). Another manifestation of the light-driven scattering dynamics was recently observed in hematite (α-Fe_2_O_3_), where resonant excitation of a 2M mode generated a zone-center magnon and induced a measurable shift in its central frequency—evidence of photoinduced magnon-spectrum renormalization previously reported only in ferromagnets.^21^

We anticipate that further advances in methods for exciting and probing finite-momentum spin waves with fs pulses will greatly expand the accessible landscape of nonlinear magnonics. By enabling strong, amplitude-driven interactions among magnons far from the Brillouin-zone center, these techniques open a route to regimes in which nonlinearities dominate the spin-wave dynamics. Such conditions are expected to support phenomena such as magnon self-focusing and the emergence of robust magnetic solitons, as well as the driven accumulation of magnons that may facilitate Bose-Einstein-like condensation in antiferromagnetic systems.

### Chiral magnons

Chiral magnons have recently emerged as a key concept in antiferromagnetic spintronics and magnonics. Their intrinsic handedness enables nonreciprocal magnon propagation, offering a promising route toward ultrafast and energy-efficient spin-based information technologies.^35^^,^^36^^,^^37^

The simplest way to create conditions for the existence of chiral magnons in an antiferromagnet is to apply magnetic field along the antiferromagnetic Néel vector. In this case, two otherwise degenerated magnon branches split, corresponding to right-handed and left-handed circularly polarized magnon bands, respectively.^38^ For the simplest case of a uniaxial antiferromagnet with easy-axis anisotropy, the chiral magnon frequencies become $\omega_{\begin{array}{c} \pm \end{array}}$:

(Equation 5) $$\omega_{\begin{array}{c} \pm \end{array}}\left(k\right)=\omega \left(k\right)\pm \gamma H\text{,}$$

where *ω*(*k*) is the frequency of the zero-field degenerate branches (see Equation 1). Note that the splitting is strongest for long-wavelength magnons, while at large wavevectors, the dispersion becomes linear, *ω*(*k*)≈*V*_0_*k*, and the degeneracy between the chiral modes is effectively restored.

Importantly, this field does not need to be external. Instead, it can be effectively generated by specific magnetic interactions allowed by the symmetry of the antiferromagnet and can give rise to additional invariants in the thermodynamic potential Φ. A particularly relevant illustration is offered by a special subclass of antiferromagnets known as altermagnets,^39^ whose symmetry permits invariants of the form ${D_{ij}M}_{i}L_{j}$, originating from the Dzyaloshinskii-Moriya interaction. This contribution is well known as the source of sublattice canting,^40^^,^^41^ and the corresponding splitting of magnon modes can, in principle, be evaluated from the effective internal magnetic field **H**_eff_ = −∂Φ/∂**M**. However, it can be shown that for the most representative altermagnets, such as MnF_2_, α-Fe_2_O_3_, and the orthoferrites, this mechanism of the splitting is essentially absent.

The altermagnetic symmetry also admits a contribution of the form $A_{ij}\frac{\partial M}{\partial x_{i}}\frac{\partial L}{\partial x_{j}}$, which is bilinear in the spatial derivatives of the magnetization and the Néel vector. This invariant is rotationally symmetric in spin space; thus, the term is of exchange origin, but anisotropic in real space, reflecting the crystal symmetry. The second-rank tensor *A*_*ij*_ has the same symmetry as the Dzyaloshinskii-Moriya tensor *D*_*ij*_, and generates an effective magnetic field:

(Equation 6) $$H_{\text{eff}}=A_{ij}\frac{\partial^{2}L}{\partial x_{i}\partial x_{j}}\text{,}$$

for which only the symmetric part of *A*_*ij*_ contributes. For rutile altermagnets such as MnF_2_ and RuO_2_, the only nonzero components are $A_{xy}=A_{yx}=\bar{A}$, giving mgagnon frequencies^42^:

(Equation 7) $$\omega_{\pm }\left(k\right)=\omega \left(k\right)\pm \gamma H_{\text{eff}}\left(k\right)\;H_{\text{eff}}\left(k\right)=\gamma \bar{A}\left|L\right|k_{x}k_{y}\text{.}$$

One can see that Equation 7 closely mirrors the structure of Equation 4, with the only difference that the effective field now depends on both the direction and the magnitude of the magnon wavevector **k**. Consequently, the resulting chiral splitting is strongly anisotropic: it vanishes for **k** oriented along the second-order symmetry axes in the basal plane and reaches its maximum for *k*_*x*_ = *k*_*y*_. We note that although Equation 7 is derived within the macroscopic approximation (*k*≪1/*a*), this expression remains valid across a broad interval of wavevectors, including those in the linear-dispersion regime $\omega_{0}/c\ll k\ll 1/a$. The emergence of such chiral magnons has recently been demonstrated numerically for RuO_2_ using *ab initio* density functional theory calculations within the pure-exchange approximation^33^ (see Figure 3C). In agreement with the phenomenological arguments above, the splitting disappears at the center and at the boundaries of the Brillouin zone. This result was later corroborated by a generalized phenomenological theory of spin dynamics incorporating nonuniform exchange interactions.^42^

It is important to emphasize that, unlike the case of a uniform magnetic field, the symmetry-generated internal field produces a significant modification of the linear-dispersion regime |**k**|≫*ω*_0_/*c*. In particular, magnons of opposite chiralities acquire different group velocities, which helps to distinguish their handedness. We anticipate that research on chiral magnons in altermagnets will greatly benefit from methods capable of exciting and detecting magnons with large finite wavevectors that reach the linear-dispersion regime, such as those discussed above. Chiral magnons constitute a highly intriguing subject for fundamental research, with significant potential impact on future information-processing technologies.

From a practical perspective, materials hosting chiral magnons offer intriguing opportunities for directional control of magnon propagation. For instance, in photonics, chiral materials have long been discussed as media in which their chirality can lock the direction of wave propagation to its polarization. As a result, such materials have functionalities allowing the realization of optical isolators or diodes, unidirectional photonic circuits, and on-chip routing.^43^ One of the obvious obstacles preventing application of chiral photonics in commercial devices is that the locking effect is still relatively small, especially when one considers optical propagation lengths at the scale of integrated photonic circuit. It is thus interesting to explore if the propagation of chiral magnons is better locked to their polarization than that of photons. Also chiral photonics for on-chip biosensing is presently another hot area of research.^44^ One can anticipate that chiral magnon-polaritons can also be employed for complementary biosensing approaches in sub-THz and THz spectral range.

### Edge magnons as proxy species of magnetic exchange

A laser pulse is among the shortest stimuli available in condensed matter physics. By selectively exciting electronic, orbital, or lattice vibrational transitions—and thereby modifying the electron wavefunctions—it is, in principle, possible to control the exchange interaction between magnetic sublattices in antiferromagnets.^45^^,^^46^^,^^47^ This opens unique and highly attractive opportunities for fundamental studies of magnetic phase transitions, where one could mimic ultrafast heating without actual thermal load or even achieve ultrafast cooling of a magnetic system by weakening or strengthening the exchange interaction.

However, understanding the dynamics of exchange coupling between spins in strongly nonequilibrium states prepared by ultrafast laser excitation is among the challenges of modern magnetism.^48^ Since the frequency of magnons at the Brillouin-zone edge is determined by the strength of the exchange interaction, these magnons can serve as proxy excitations for tracking its time-dependent evolution. Time-resolved Raman measurements of the 2M mode in antiferromagnetic perovskite KNiF_3_ reveal a photoinduced shift in its frequency as a function of pump-probe delay (see Figure 3C), directly reflecting the optically induced changes of the exchange interaction.^34^

The approaches discussed here may also extend to multiferroic and magnetoelectric materials,^49^ where coupling between magnetic and ferroelectric order enables access to electromagnons and magnetoelectric excitations at finite momentum.^50^ Moreover, light-driven excitation of coherent high-*k* antiferromagnetic magnons with reasonable large *Q*-factors provides a natural platform for exploring nonclassical THz magnon states, including squeezed and entangled magnon pairs.^51^^,^^52^ Advances in nanoscale THz magnonics may also enable significant boosting of operational frequencies in emerging hybrid quantum architectures based on magnonics.^53^

## Conclusions

Studying magnons with nanometer wavelengths and THz frequencies offers an opportunity to explore the genuine quantum, and therefore often counterintuitive, nature of magnetism. Recent advances in fs laser technology have enabled relatively simple and accessible methods for generating coherent magnons with these characteristics in antiferromagnetic materials, as well as for detecting them in the coherent regime. Antiferromagnets themselves provide a fascinating platform for investigating the dynamics of propagating magnons, with promising implications for future information-processing technologies and opening new avenues in magnetic research. Looking ahead, these capabilities could be combined with on-chip mode-locked semiconductor lasers^54^^,^^55^ to realize ultrafast magnonic devices, paving the way toward THz-rate logic operations. By selectively exciting and detecting coherent THz magnons in engineered magnonic waveguides, for example, created by selective ion implantation^56^ or patterned antiferromagnetic nanostructures, such architectures may enable on-chip data processing at unprecedented speeds while also offering a platform for exploring nonclassical magnon states, including squeezed and entangled pairs, for hybrid quantum technologies.

## Acknowledgments

This work has received funding from the 10.13039/501100000781European Research Council (ERC) under ERC grant agreement nos. 101078206 (ASTRAL) and 101054664 (SPARTACUS). The work has also been supported by “Materials for the Quantum Age” (QuMat, registration number 024.005.006), which is part of the Gravitation program financed by the Dutch Ministry of Education, Culture and Science (OCW).

## Declaration of interests

The authors declare no competing interests.
